# Cardiovascular magnetic resonance imaging markers of ageing: a multi-centre, cross-sectional cohort study

**DOI:** 10.1093/ehjopen/oeaf032

**Published:** 2025-05-02

**Authors:** Hosamadin S Assadi, Xiaodan Zhao, Gareth Matthews, Rui Li, Jordi Broncano Cabrero, Bahman Kasmai, Samer Alabed, Javier Royuela Del Val, Hilmar Spohr, Yashoda Gurung-Koney, Nay Aung, Sunil Nair, Andrew J Swift, Vassilios S Vassiliou, Liang Zhong, Abdallah Al-Mohammad, Rob J van der Geest, Peter P Swoboda, Sven Plein, Pankaj Garg

**Affiliations:** Department of Cardiovascular and Metabolic Health, Norwich Medical School, University of East Anglia, Norwich Research Park, Norwich NR4 7UQ, Norfolk, UK; Norfolk and Norwich University Hospitals NHS Foundation Trust, Norwich NR4 7UY, Norfolk, UK; National Heart Research Institute Singapore, National Heart Centre Singapore, 5 Hospital Drive, Singapore 169609, Singapore; Department of Cardiovascular and Metabolic Health, Norwich Medical School, University of East Anglia, Norwich Research Park, Norwich NR4 7UQ, Norfolk, UK; Norfolk and Norwich University Hospitals NHS Foundation Trust, Norwich NR4 7UY, Norfolk, UK; Department of Cardiovascular and Metabolic Health, Norwich Medical School, University of East Anglia, Norwich Research Park, Norwich NR4 7UQ, Norfolk, UK; Norfolk and Norwich University Hospitals NHS Foundation Trust, Norwich NR4 7UY, Norfolk, UK; Cardiothoracic Imaging Unit, Hospital San Juan de Dios, Ressalta, HT Medica, Avenida el Brillante No. 36, 14012 Córdoba, Spain; Department of Cardiovascular and Metabolic Health, Norwich Medical School, University of East Anglia, Norwich Research Park, Norwich NR4 7UQ, Norfolk, UK; Norfolk and Norwich University Hospitals NHS Foundation Trust, Norwich NR4 7UY, Norfolk, UK; Department of Infection, Immunity and Cardiovascular Disease, University of Sheffield, Sheffield S10 2RX, UK; Cardiothoracic Imaging Unit, Hospital San Juan de Dios, Ressalta, HT Medica, Avenida el Brillante No. 36, 14012 Córdoba, Spain; Norfolk and Norwich University Hospitals NHS Foundation Trust, Norwich NR4 7UY, Norfolk, UK; Norfolk and Norwich University Hospitals NHS Foundation Trust, Norwich NR4 7UY, Norfolk, UK; Barts Heart Centre, St Bartholomew’s Hospital, Barts Health NHS Trust, West Smithfield, London EC1A 7BE, UK; Norfolk and Norwich University Hospitals NHS Foundation Trust, Norwich NR4 7UY, Norfolk, UK; National Heart Research Institute Singapore, National Heart Centre Singapore, 5 Hospital Drive, Singapore 169609, Singapore; Department of Cardiovascular and Metabolic Health, Norwich Medical School, University of East Anglia, Norwich Research Park, Norwich NR4 7UQ, Norfolk, UK; Norfolk and Norwich University Hospitals NHS Foundation Trust, Norwich NR4 7UY, Norfolk, UK; National Heart Research Institute Singapore, National Heart Centre Singapore, 5 Hospital Drive, Singapore 169609, Singapore; Duke-NUS Medical School, National University of Singapore, Singapore 169857, Singapore; Department of Biomedical Engineering, National University of Singapore, Singapore 117583, Singapore; Department of Infection, Immunity and Cardiovascular Disease, University of Sheffield, Sheffield S10 2RX, UK; Department of Radiology, Division of Image Processing, Leiden University Medical Center, Leiden 2333 ZA, The Netherlands; Division of Biomedical Imaging, Leeds Institute of Cardiovascular and Metabolic Medicine, University of Leeds, Leeds LS2 9JT, UK; Division of Biomedical Imaging, Leeds Institute of Cardiovascular and Metabolic Medicine, University of Leeds, Leeds LS2 9JT, UK; Department of Cardiovascular and Metabolic Health, Norwich Medical School, University of East Anglia, Norwich Research Park, Norwich NR4 7UQ, Norfolk, UK; Norfolk and Norwich University Hospitals NHS Foundation Trust, Norwich NR4 7UY, Norfolk, UK

**Keywords:** Ageing, Diabetes mellitus, Hypertension, Magnetic resonance imaging (cine), Obesity

## Abstract

**Aims:**

Cardiac ageing involves a series of anatomical and physiological changes contributing to a decline in overall performance. Cardiac magnetic resonance (CMR) provides comprehensive structural and functional assessment for detecting age-related cardiovascular remodelling. We aimed to develop a fully automated CMR model to predict functional heart age.

**Methods and results:**

This international, multi-centre, retrospective observational study enrolled 191 healthy individuals with normal body mass index (BMI), free of metabolic, cardiovascular, and respiratory disease as the derivation cohort. Left atrial (LA) end-systolic volume and LA ejection fraction were selected for the final model. The model was validated on 366 patients with BMI >25 kg/m^2^ and one or more comorbidities [hypertension, diabetes mellitus (DM), atrial fibrillation (AF), and obesity]. In healthy individuals [median age: 34 years, 105 (55%) female], CMR-derived functional heart age was similar to the chronological age [bias: 0.05%, 95% confidence interval (CI): 9.56–9.67%, *P* = 0.993]. In the validation cohort [median age: 53 years, 157 (43%) female], CMR-derived functional heart age was 4.6 years higher than chronological age (95% CI: 1.6–7.6 years, *P* = 0.003). Cardiac magnetic resonance-derived functional heart age was significantly higher in patients with hypertension (*P* < 0.001), DM (*P* < 0.001), and AF (*P* < 0.001) than age-matched healthy controls. Moreover, CMR-derived functional heart age was higher than the chronological age in obesity Class I (*P* = 0.07), obesity Class II (*P* = 0.11), and obesity Class III (*P* < 0.001).

**Conclusion:**

This study highlights the time course of structural and physiological changes in the heart during healthy and unhealthy ageing. We propose simple equations that should help communicate subtle changes in heart assessment with ageing.

**Registration:**

ClinicalTrials.gov: NCT05114785

## Graphical Abstract

**Graphical Abstract:**
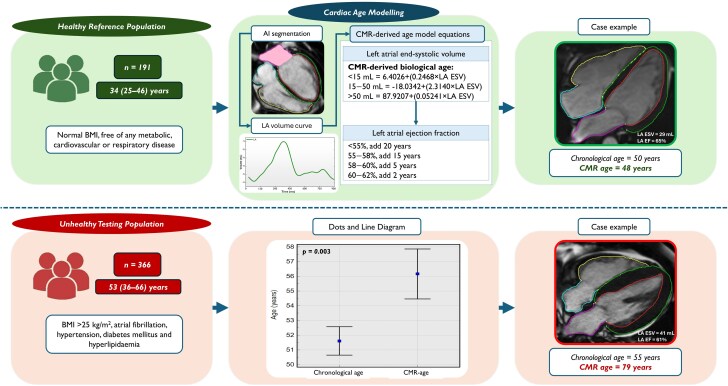

## Introduction

Cardiovascular disease (CVD) poses a significant health challenge, particularly with the ageing global population. In 2022, more than 19 million people died from CVD, with ischaemic heart disease and stroke accounting for 85% of the total age-standardized death rate of CVD.^1^ As people age, the prevalence of CVD increases, placing a growing burden on healthcare systems. Pre-mature cardiovascular ageing is described as a drop in cardiovascular reserve and stress resistance, alongside significant changes in gene expression related to decreased stress response, quality control, and mitochondrial pathways.^2^ Modifiable risk factors such as hypertension, diabetes mellitus (DM), and obesity accelerate the cardiovascular ageing process. Chronic high blood pressure triggers vascular stiffening and left ventricular (LV) hypertrophy, which are hallmarks of accelerated cardiovascular ageing and major adverse cardiac events.^3,4^ Poorly controlled DM accelerates cardiovascular ageing by inducing hyperglycaemia-induced oxidative stress and inflammation, initiating endothelial dysfunction and atherosclerosis.^5^ Obesity accelerates cardiac ageing through various interrelated mechanisms, including mitochondrial dysfunction, increased oxidative stress, and telomere shortening, leading to compromised cardiac function and increased risk of heart failure (HF).^6^

Cardiac magnetic resonance (CMR) imaging is recognized as the reference standard for non-invasive cardiac functional and volumetric assessment,^7^ offering comprehensive means to study age-related cardiovascular changes. Cardiac magnetic resonance provides detailed information on cardiac structure and function, including myocardial fibrosis, ventricular dimensions, and systolic and diastolic function, without exposing patients to ionizing radiation.^3,8^ Cardiac magnetic resonance can provide valuable insights into granular details of chamber size, geometry, function, and deformation, which is invaluable for detecting pre-mature heart ageing in patients with hypertension, DM, and obesity.^9–11^

Ageing is a multi-faceted process linked to many disease states, and it can be consistent over time or variable, involving sudden shifts or accelerations at specific points in life.^12^ Understanding the physiological impact of non-modifiable risk factors like age and modifiable risk factors are crucial in addressing these aspects through lifestyle changes and medical interventions. This can significantly slow the progression of cardiovascular ageing and reduce the risk of associated diseases. Despite the numerous and inexpensive therapies and lifestyle modifications for cardiovascular risk reduction, many patients might decline while on preventative treatments due to an incomplete comprehension of risk.^13,14^ Such factors might disproportionately affect certain ethnic, socioeconomic, and educational backgrounds. Underuse of preventative treatments has significant impacts on patient and population levels.^15,16^ Previous studies have emphasized the importance of communicating risk concepts simply and in a patient-centred way.^17^ Pooled cohort studies have been used to estimate risk, but there remains a discrepancy in how patients interpret the results, mainly as the results are statistical rather than individualized.^18^ Age is a very tangible concept for most patients to understand, and physicians could use a discrepancy between the individual patient’s CMR-derived functional heart age and the chronological age to highlight the need for risk factor modification. Even though ageing concepts have adequately been described in radiomics and genomics,^19,20^ no simple CMR model exists to predict chronological age. We hypothesize that chronological age can be modelled using CMR-derived parameters collected as part of standard CMR assessment.

This study aims to (i) develop a CMR model to estimate functional heart age using mathematical modelling and physiological aspects, (ii) investigate the relationship between cardiovascular risk factors and pre-mature cardiovascular ageing characteristics, and (iii) detail the structural and functional alterations in the heart linked to pre-mature cardiovascular ageing.

## Methods

### Study cohort

In this multi-centre, multi-vendor, international, retrospective observational, cross-sectional cohort study, we included 563 subjects enrolled between January 2015 and December 2023 from 5 centres across the globe. This included 95 participants from the PREFER-CMR registry (NCT05114785) in Norfolk and Norwich University Hospitals NHS Foundation Trust, United Kingdom, 33 participants from Leeds Teaching Hospitals NHS Foundation Trust, United Kingdom, 15 participants from Sheffield Teaching Hospitals NHS Foundation Trust, United Kingdom, 255 participants from HT Médica in Cordoba, Spain and 165 participants from a prospective multi-centre registry (NCT03217240) in Singapore, Singapore.

The chronological age was defined as the age determined by the amount of time elapsed from birth to the current indexed date. The CMR functional heart age was defined as the age determined by an estimate from the parameters with the most sensitive morphological and functional changes with ageing measured by CMR in healthy subjects without any known cardiovascular risk factors from 10 to 85 years of age. The cohort was stratified into seven groups according to their age: Group 1 (*n* = 43, age range: 10–19 years); Group 2 (*n* = 82, age range: 20–29 years); Group 3 (*n* = 99, age range: 30–39 years); Group 4 (*n* = 92, age range: 40–49 years); Group 5 (*n* = 80, age range: 50–59 years); Group 6 (*n* = 89, age range: 60–69 years); and Group 7 (*n* = 78, age range: 70–85 years). Also, we divided our cohort based on their health status into healthy (*n* = 191) and unhealthy (*n* = 366). Finally, for preliminary external validation, we utilized a cohort of 25 healthy individuals from Norfolk and Norwich University Hospitals NHS Foundation Trust, United Kingdom, and Leeds Teaching Hospitals NHS Foundation Trust, United Kingdom. This study complies with the Declaration of Helsinki. Data acquisition and handling were authorized by the National Research Ethics Service (Ref.: 21/NE/0149, Ref.: 18/NE/0186, and Ref.: 12/YH/0169) in the UK, and the International Review Board (Ref.: 2016/3017) in Singapore. Written informed consent was obtained from all participants.

**Figure 1:**
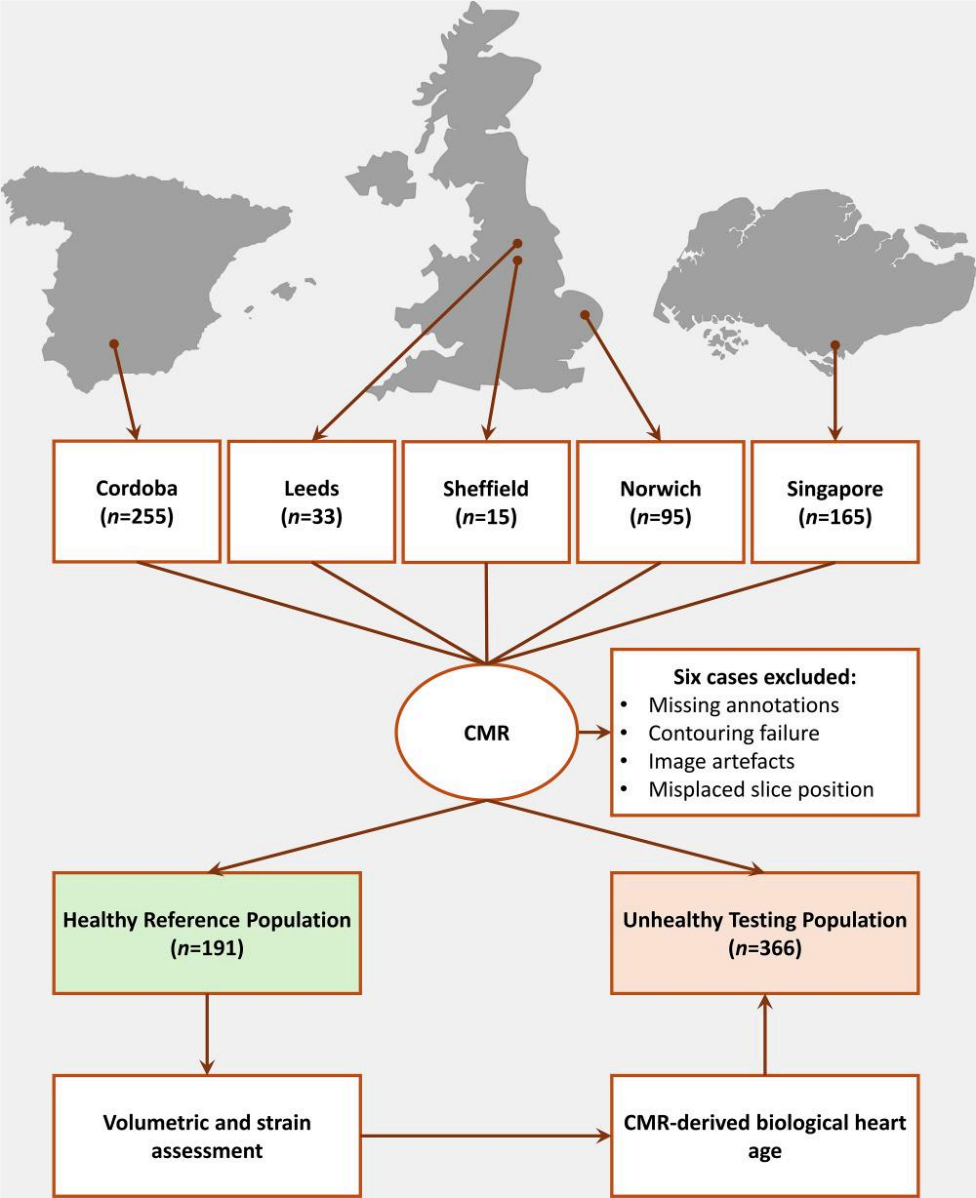
Study flow chart. Flow chart illustrating the process of deriving predictive models for functional heart age using CMR data. CMR, cardiac magnetic resonance.

Patient and public involvement statement is presented in Supplementary material online, *Methods*. The study adhered to the strengthening the reporting of observational studies in epidemiology guidelines.^21^ A flow chart illustrating the recruitment process and the steps taken to develop and test the cardiac-age model is shown in *Figure 1*.

### Healthy cohort

The inclusion criteria for the healthy cohort were individuals with normal body mass index (BMI <25 kg/m^2^) and free of any metabolic, cardiovascular, or respiratory disease. Other inclusion criteria included individuals with good-quality scans for segmentation.

### Unhealthy cohort

The inclusion criteria for the unhealthy cohort were patients with one or more comorbidities: overweight and obese (BMI >25 kg/m^2^), atrial fibrillation (AF), hypertension, DM, and hyperlipidaemia. Moreover, only individuals with good-quality scans for segmentation were included.

### Exclusion criteria

The exclusion criteria for all subjects were inability to lie flat, LV ejection fraction (EF) <50%, pregnancy, incompatible devices or implants, or any other contraindication to CMR, including allergy to contrast, claustrophobia, and chronic kidney disease Stages IV and V (estimated glomerular filtration rate <30 mL/min/1.73 m^2^).

### Cardiac magnetic resonance protocol for acquisition, image analysis, and model development

The CMR protocol, including cine acquisition parameters for each centre, is provided in Supplementary material online, *Methods* and *Table S1*.

All image analyses were performed using the recently developed fully convolutional neural network artificial intelligence (AI) integrated batch processing function in MASS research software (MASS, Version 2023-EXP, Leiden University Medical Centre, Leiden, The Netherlands).^22^ The contours generated include endocardial borders for the LV, right ventricle (RV), left atrial (LA), and right atrium (RA) and epicardial borders for the LV in all cardiac phases on the four-chamber cine. All four-chamber volumes were estimated using the area-length method. Papillary muscles and trabeculations were included in the volume calculation. End-diastolic volume (EDV), a measure of global diastolic function, was defined as the volume of blood in the ventricle at the end of the filling phase, while end-systolic volume (ESV), a measure of systolic function, was defined as the volume of blood in the ventricle at the end of the ejection phase, just before ventricular relaxation. Stroke volume (SV) was defined as the volume of blood ejected during systole (SV = EDV − ESV), and cardiac output (CO) was defined as the amount of blood ejected per minute (CO = SV × heart rate). Ejection fraction was defined as the ratio of SV to EDV (EF = SV/EDV × 100). Left ventricle peak ejection rate (PER) was defined as the maximum rate at which blood is ejected from the LV during systole, and peak filling rate (PFR) as the maximum rate at which the LV fills with blood during diastole. Left ventricle mass was calculated from myocardial volume and tissue density. Global longitudinal strain (GLS), a sensitive marker of myocardial deformation, was measured as the percentage change in the length of the myocardial fibres longitudinally. A visual assessment of the AI-generated segmentations and time-resolved volume curves throughout all cardiac phases was carried out for quality control. Six of 563 (1%) cases with either missing annotations, substantial contouring failure of cardiac chambers, image artefacts, or misplaced slice position were excluded.

### Cardiac-age model development

A central illustration demonstrating an overview of the study flow chart is presented in the *Graphical abstract*. We aimed to ensure that the statistical power and reliability of the findings aligned with established statistical principles. To provide adequate data for model development without overfitting, we included 191 healthy participants in the derivation cohort. For validation, we included 366 unhealthy patients with the conditions of interest to ensure the model's generalizability and accuracy in a real-world setting, as the larger sample helps to account for the increased variability and ensures the stability of the model's performance metrics.^23^ We employed iterative methodologies to develop and validate the functional heart age model, using the age of healthy controls as the reference standard. Missing data were avoided for model development, and no interpolation techniques were used to account for missing data.

The Spearman coefficient of rank correlation was computed to explore the correlation between chronological age and all CMR metrics in the derivation healthy cohort. When variables exhibited high collinearity and similar physiological significance, the variable with the most physiological relevance was chosen for further analysis. Importantly, we also visualized how each variable (median values) adapted in every decade of life. If the variable demonstrated nonlinear complex behaviour, we avoided including that variable further in the development of the equation. Provisional validation work was done to investigate if the cardiac chamber variable adapted positively or negatively in the unhealthy cohort to reduce the chance of either over or underestimation of cardiac age—this process was repeated to achieve a sensible fit. Preference was given to the geometrical model, which offers the advantage of being applied to any picture archiving and communication system, digital imaging, and communications in medicine system without needing specialized software for post-processing cardiac cine images.

Once key variables were identified, even if they predominantly behaved linearly due to the natural sigmoid behaviour, we further developed interpolated data to account for extremes instead of choosing nonlinear regression. An internal validation procedure was performed iteratively to check for agreements. Interpolated data at the extremes of age are presented in Supplementary material online, *Table S2*. For some variables, we used thresholds to correct the linearly modelled age. This was done to adjust the age-related steep decrease at certain decades in life, which allowed the final equation to have the least bias.

Final internal validation was done using Bland–Altman analysis to investigate systematic differences and Spearman's coefficient of rank correlation to establish correlation. All model development adhered to the transparent reporting of a multi-variable prediction model for individual prognosis or diagnosis guidelines.^24^

### Statistical analysis

Statistical analyses were conducted using SPSS Statistics (IBM, Chicago, USA, version 29) and MedCalc (MedCalc Software, Ostend, Belgium, version 22.009). Normal distribution was tested using the Shapiro–Wilk test. Continuous variables were summarized using median with inter-quartile range (IQR). A two-sample independent *t*-test was used to compare continuous variables. Associations between continuous variables were evaluated using the Spearman coefficient of rank correlation (*ρ*) for non-parametric data. Categorical data were expressed as frequencies and percentages. To compare continuous variables, the Kruskal–Wallis (K–W) one-way analysis of variance test was used for non-parametric data with *post hoc* pair-wise comparisons if the K–W test was significant. Univariate and multi-variable stepwise regression analyses were performed to analyse the predictors of ageing. Only the variables with significant associations in univariate analyses were used as inputs for subsequent multi-variate linear regression analyses. Unless otherwise indicated, all statistical tests were two-tailed, and significance was defined as a *P*-value <0.05.

## Results

### Study population

The demographic data and CMR characteristics of the healthy and unhealthy groups are presented in *Tables 1* and *2*. The healthy group’s median (IQR) age was 34 years (25–46 years), and 105 of 191 (55%) subjects were female. The unhealthy group had a median (IQR) age of 53 years (36–66 years), and 157 of 366 (43%) were female. Body mass index was significantly lower in the healthy group [median (IQR) 21 (20–23 kg/m^2^) vs. 27 (25–31 kg/m^2^), *P* < 0.001]. In the unhealthy group, 54 (15%) had hyperlipidaemia, 79 (22%) were hypertensive, 41 (11%) were diabetic, 23 (6%) had AF, and 22 (6%) had a previous history of myocardial infarction (all *P* < 0.001). Overall, the unhealthy group had higher median left and right heart EDV, ESV, and SV. Left atrial EF was significantly higher in the healthy group, while LV EF, RA EF, and RV EF (62% for both) were comparable. A subanalysis comparing European vs. non-European is demonstrated in Supplementary material online, *Table S3*.

**Table 1:** Study demographics of healthy and unhealthy individuals

Demographics	Healthy (*n* = 191)	Unhealthy (*n* = 366)	*P*-value
Age, years	34 (25–46)	53 (36–66)	<0.0001
Female sex, *n* (%)	105 (55)	157 (43)	0.007
Body mass index, kg/m^2^	21 (20–23)	27 (25–31)	<0.0001
Hyperlipidaemia, *n* (%)	0 (0)	54 (15)	<0.0001
Hypertension, *n* (%)	0 (0)	79 (22)	<0.0001
Diabetes mellitus, *n* (%)	0 (0)	41 (11)	<0.0001
Atrial fibrillation, *n* (%)	0 (0)	23 (6)	<0.0001
Myocardial infarction, *n* (%)	0 (0)	22 (6)	<0.001

Data are given as median (25th–75th percentile), a Mann–Whitney test.

Healthy includes individuals with normal BMI < 25 kg/m^2^ and free of any metabolic, cardiovascular, and respiratory disease. Unhealthy includes patients with one or more comorbidities: overweight and obese (BMI >25 kg/m^2^), atrial fibrillation, hypertension, diabetes mellitus, and hyperlipidaemia.

**Table 2:** Cardiac magnetic resonance characteristics of healthy and unhealthy individuals

	Healthy (*n* = 191)	Unhealthy (*n* = 366)	*P*-value
Left heart			
Left atrial end-diastolic volume, mL	68 (53–81)	75 (61–94)	**<0**.**0001**
Left atrial end-systolic volume, mL	23 (17–30)	28 (21–37)	**<0**.**0001**
Left atrial stroke volume, mL	43 (34–52)	46 (37–55)	**0**.**011**
Left atrial ejection fraction, %	65 (60–69)	62 (56–67)	**<0**.**001**
Left atrial global longitudinal strain, %	−28 (−33 to −24)	−26 (−30 to −20)	**<0**.**0001**
Left ventricular end-diastolic volume, mL	126 (109–150)	138 (120–164)	**<0**.**0001**
Left ventricular end-systolic volume, mL	47 (40–59)	52 (41–65)	**0**.**014**
Left ventricular stroke volume, mL	78 (67–88)	86 (74–102)	**<0**.**0001**
Left ventricular mass, g	85 (69–108)	111 (92–131)	**<0**.**0001**
Left ventricular ejection fraction, %	62 (59–65)	63 (58–67)	0.107
Left ventricular peak ejection rate, mL/s	382 (318–447)	414 (350–495)	**<0**.**001**
Left ventricular peak filling rate, mL/s	448 (385–573)	464 (373–582)	0.804
Left ventricular cardiac output, mL/min	4847 (4119–5679)	5579 (4731–6579)	**<0**.**0001**
Left ventricular global longitudinal strain, %	−21 (−24 to −19)	−22 (−24 to −19)	0.763
Right heart			
Right atrial end-diastolic volume, mL	65 (54–75)	75 (57–93)	**<0**.**0001**
Right atrial end-systolic volume, mL	29 (23–38)	34 (25–46)	**<0**.**001**
Right atrial stroke volume, mL	34 (27–41)	38 (30–48)	**<0**.**001**
Right atrial ejection fraction, %	53 (48–59)	52 (47–58)	0.246
Right atrial global longitudinal strain, %	−28 (−31 to −24)	−27 (−30 to −23)	**0**.**029**
Right ventricular end-diastolic volume, mL	70 (57–86)	82 (65–101)	**<0**.**0001**
Right ventricular end-systolic volume, mL	27 (20–35)	31 (22–41)	**<0**.**001**
Right ventricular stroke volume, mL	43 (35–53)	50 (40–61)	**<0**.**0001**
Right ventricular ejection fraction, %	62 (58–67)	62 (57–67)	0.790
Right ventricular cardiac output, mL/min	2644 (2091–3391)	3230 (2539–4014)	**<0**.**0001**
Right ventricular global longitudinal strain, %	−32 (−36 to −29)	−31 (−35 to −27)	0.051

Data are given as median (25th–75th percentile), a Mann–Whitney test.

Bold values denote statistical significance. Healthy includes individuals with normal BMI < 25 kg/m^2^ and free of any metabolic, cardiovascular, and respiratory disease. Unhealthy includes patients with one or more comorbidities: overweight and obese (BMI >25 kg/m^2^), atrial fibrillation, hypertension, diabetes mellitus, and hyperlipidaemia.

### Cardiac magnetic resonance parameters associated with ageing

**Figure 2:**
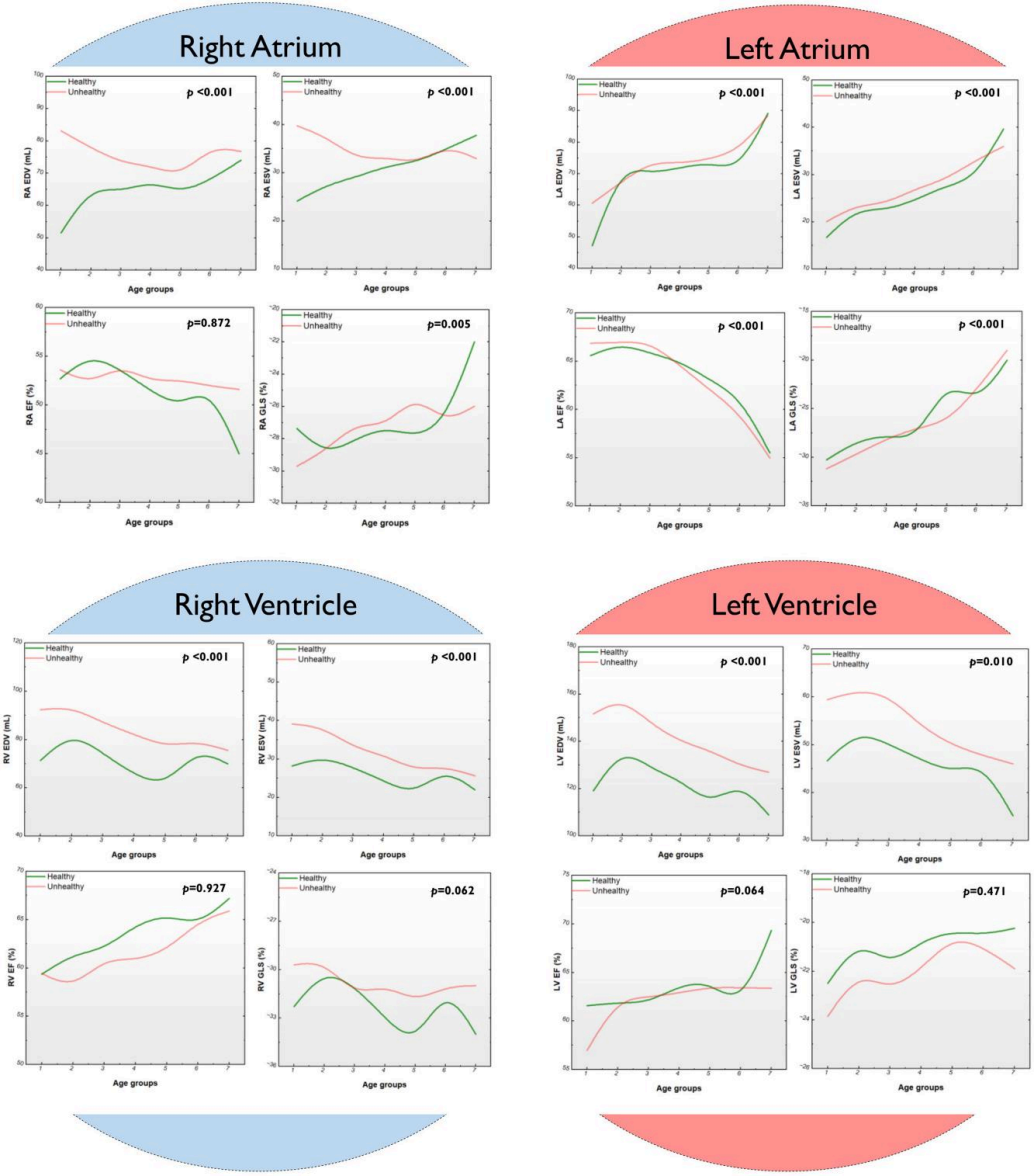
Cardiac magnetic resonance parameters associated with healthy and unhealthy ageing. Cardiac magnetic resonance imaging parameters of the four chambers of the heart associated with healthy (green line) and unhealthy (red line) ageing and their differences in different decades of life. EDV, end-diastolic volume; GLS, global longitudinal strain; LV, left ventricle; RA, right atrium; RV, right ventricle, and other abbreviations as in *Figure 1*.

**Figure 3:**
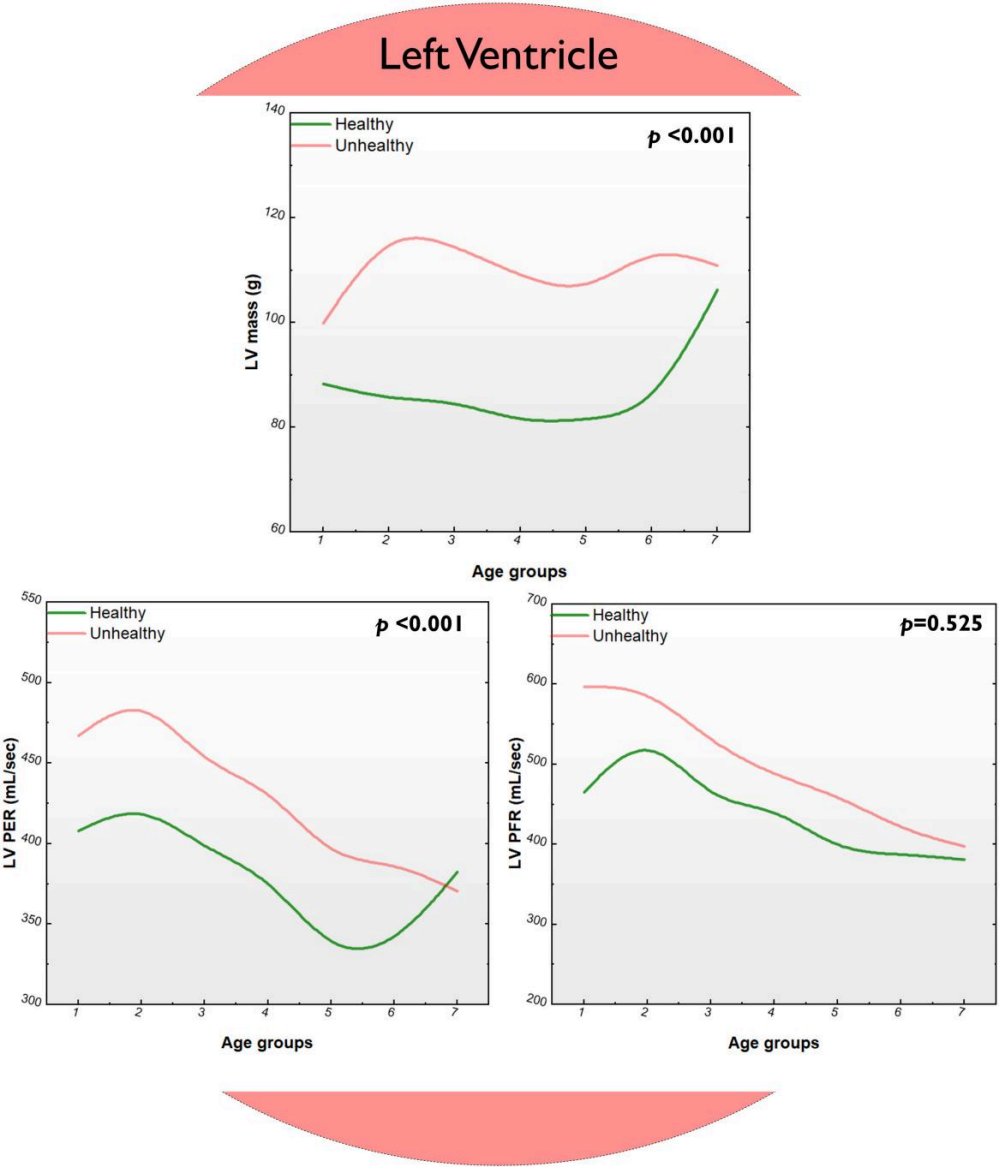
Other cardiac magnetic resonance parameters associated with healthy and unhealthy ageing. Cardiac magnetic resonance imaging parameters in the left ventricle associated with healthy (green line) and unhealthy (red line) ageing and their differences in different decades of life. LV, left ventricle; PER, peak ejection rate; PFR, peak filling rate.

Left atrial volumetric indices were comparable across all eight age groups between healthy and unhealthy cohorts. The unhealthy cohort had significantly higher LV EDV, LV ESV, LV mass, LV PER, and LV PFR than the healthy subjects. In the right heart, RA EDV, RV EDV, RV ESV, and RV GLS were significantly higher, and RV EF was significantly lower in the unhealthy group across all ages (*Figures 2* and *3*).

### Cardiac magnetic resonance parameters to predict chronological age

In the healthy group, LA EDV and LA ESV significantly increased with healthy ageing (*P* < 0.01). Also, LA EF and LV PFR decreased significantly with ageing (*P* < 0.01). Moreover, certain right heart parameters, including RA EDV, RA ESV, and RV EF, correlated positively, while RV ESV correlated negatively with healthy ageing (all *P* < 0.01; see Supplementary material online, *Table S4*).

### Cardiac magnetic resonance-derived functional heart age equation

The CMR-functional heart age equation was exclusively derived using data from the healthy cohort (*n* = 169). From the CMR functional parameters that displayed significant correlation with age in this cohort, two variables, LA ESV and LA EF, were entered into the final model. Left atrial ESV had the most linear time curve in both healthy and unhealthy cohorts. Left ventricle PFR was not selected as it was positively compensated in the unhealthy cohort. We used three regression equations for LA ESV to factor in the extremes—(<15, 15–50, and >50 mL). For LA ESV 15–50 mL, a strong correlation coefficient of determination (*R*^2^) was found with chronological age (*R*^2^ = 0.829, *P* = 0.002). The correlation remained strong for higher extremes with interpolated blunted age (LA ESV >50 mL: *R*^2^ = 0.938, *P* = 0.032). Similar results were noted for lower extremes (LA ESV <15 mL: *R*^2^ = 0.970, *P* = 0.002).

The final regression equations were as follows:

1. If LA ESV <15 mL: CMR-derived functional heart age = 6.4026 + (0.2468 × LA ESV).
2. If LA ESV 15–50 mL: CMR-derived functional heart age = −18.0342 + (2.3140 × LA ESV).
3. If LA ESV >50 mL: CMR-derived functional heart age = 87.9207 + (0.05241 × LA ESV).

Then, LA EF was used to correct for systematic bias by the following thresholds:

1. If LA EF <55%, add 20 years.
2. If LA EF 55–58%, add 15 years.
3. If LA EF 58–60%, add 5 years.
4. If LA EF is 60–62%, add 2 years.

Preliminary external validation results of the above equations in 25 healthy individuals are presented in the Supplementary material online, *Results*, *Table S5* and *Figure S1*.

### Healthy cohort

In the healthy cohort, the correlation coefficient of the CMR-derived functional heart age model with the chronological age was [*ρ* = 0.329, 95% confidence interval (CI): 0.196–0.450, *P* < 0.001]. On Bland–Altman analysis, the CMR-derived age was similar to chronological age (bias: 0.05%, 95% CI: −9.56–9.67%, *P* = 0.993).

### Unhealthy cohort

In the unhealthy cohort, the CMR-derived functional heart age was significantly higher than the chronological age by 4.6 years (95% CI: 1.6–7.6 years, *P* = 0.003; *Figure 1*). The correlation coefficient of CMR-derived functional heart age with chronological age was (*ρ* = 0.448, 95% CI: 0.362–0.526, *P* < 0.001). On Bland–Altman analysis, the CMR-derived functional heart age was higher than the chronological age (bias: 3.73%, 95% CI: −2.76–10.22%, *P* = 0.259).

### Cardiac magnetic resonance-derived functional heart age and body mass index

We divided our cohort into five groups according to their BMI as defined by the World Health Organization criteria: normal (if BMI <25 kg/m^2^), overweight (BMI 25–29.9 kg/m^2^), obesity Class I (BMI 30–34.9 kg/m^2^), obesity Class II (BMI 35–39.9 kg/m^2^), and obesity Class III (BMI ≥40 kg/m^2^).

In a subgroup analysis comparing healthy individuals (BMI <25 kg/m^2^) with the three obesity classes, we observed significant differences in LA EDV and LA ESV, and these differences rose with BMI levels (*P* < 0.0001; *Table 3*).

**Table 3:** Cardiac magnetic resonance characteristics of healthy and obese individuals as per the World Health Organization classification

	Healthy (*n* = 191)	Obesity Class I (*n* = 66)	Obesity Class II (*n* = 34)	Obesity Class III (*n* = 7)	*P*-value
Left heart					
Left atrial end-diastolic volume, mL	68 (53–81)	83 (70–100)*	89 (62–106)*	110 (106–137)*,**,***	**<0**.**0001**
Left atrial end-systolic volume, mL	23 (17–30)	31 (23–51)*	33 (24–51)*	54 (44–79)*,**,***	**<0**.**0001**
Left atrial stroke volume, mL	43 (34–52)	48 (37–58)*	49 (38–59)	57 (53–67)*	**0**.**007**
Left atrial ejection fraction, %	65 (60–69)	61 (52–67)*	60 (52–64)*	49 (32–60)*,**	**<0**.**0001**
Left atrial global longitudinal strain, %	−28 (−33 to −24)	−25 (−29 to −16)*	−23 (−30 to −18)*	−17 (−23 to −9)*,**	**<0**.**0001**
Left ventricular end-diastolic volume, mL	126 (109–150)	136 (119–167)*	140 (128–164)*	141 (119–205)	**<0**.**001**
Left ventricular end-systolic volume, mL	47 (40–59)	53 (41–64)	48 (40–67)	56 (47–82)	0.121
Left ventricular stroke volume, mL	78 (67–88)	86 (77–102)*	93 (83–109)*	96 (73–123)*	**<0**.**0001**
Left ventricular mass, g	85 (69–108)	111 (92–130)*,***	130 (111–148)*,**	142 (120–175)*,**	**<0**.**0001**
Left ventricular ejection fraction, %	62 (59–65)	63 (58–68)	66 (61–69)*	59 (58–67)	**0**.**039**
Left ventricular peak ejection rate, mL/sec	382 (318–447)	450 (337–516)*	423 (389–494)*	464 (403–585)*	**<0**.**0001**
Left ventricular peak filling rate, mL/sec	448 (385–573)	465 (363–597)	474 (356–592)	500 (431–776)	0.834
Left ventricular cardiac output, mL/min	4847 (4119–5679)	5834 (5153–6937)*	6177 (4876–7948)*	7064 (5221–7331)*	**<0**.**0001**
Left ventricular global longitudinal strain, %	−21 (−24 to −19)	−22 (−26 to −20)	−23 (−26 to −17)	−21 (−25 to −17)	0.159
Right heart					
Right atrial end-diastolic volume, mL	65 (54–75)	76 (52–96)*	85 (64–107)*	99 (83–107)*	**<0**.**0001**
Right atrial end-systolic volume, mL	29 (23–38)	33 (21–45)	38 (33–54)*	42 (36–72)*	**<0**.**001**
Right atrial stroke volume, mL	34 (27–41)	39 (30–51)*	43 (31–56)*	44 (29–53)	**0**.**013**
Right atrial ejection fraction, %	53 (48–59)	52 (47–60)	51 (44–57)	55 (35–58)	0.762
Right atrial global longitudinal strain, %	−28 (−31 to −24)	−26 (−31 to −22)*	−26 (−30 to −21)	−21 (−28 to −12)*	**0**.**019**
Right ventricular end-diastolic volume, mL	70 (57–86)	83 (65–98)*	93 (78–115)*	96 (91–121)*	**<0**.**0001**
Right ventricular end-systolic volume, mL	27 (20–35)	29 (65–98)	35 (28–52)*	37 (36–53)*,**	**<0**.**001**
Right ventricular stroke volume, mL	43 (35–53)	53 (40–62)*	57 (49–72)*	66 (45–75)*	**<0**.**0001**
Right ventricular ejection fraction, %	62 (58–67)	63 (60–68)	61 (56–65)	60 (51–66)	0.115
Right ventricular cardiac output, mL/min	2644 (2091–3391)	3602 (2691–4405)*	4030 (2969–5004)*	3775 (3150–5079)*	**<0**.**0001**
Right ventricular global longitudinal strain, %	−32 (−36 to −29)	−31 (−36 to −28)	−30 (−35 to −25)	−29 (−31 to −23)	0.089

Data are given as median (25th–75th percentile).

Bold values denote statistical significance. Healthy includes individuals with normal BMI < 25 kg/m^2^ and free of any metabolic, cardiovascular and respiratory disease.

^*^ *P* < 0.05 compared with healthy.

^**^ *P* < 0.05 compared with obesity Class I.

^**^*P < 0.05 compared with obesity Class II.

**Figure 4:**
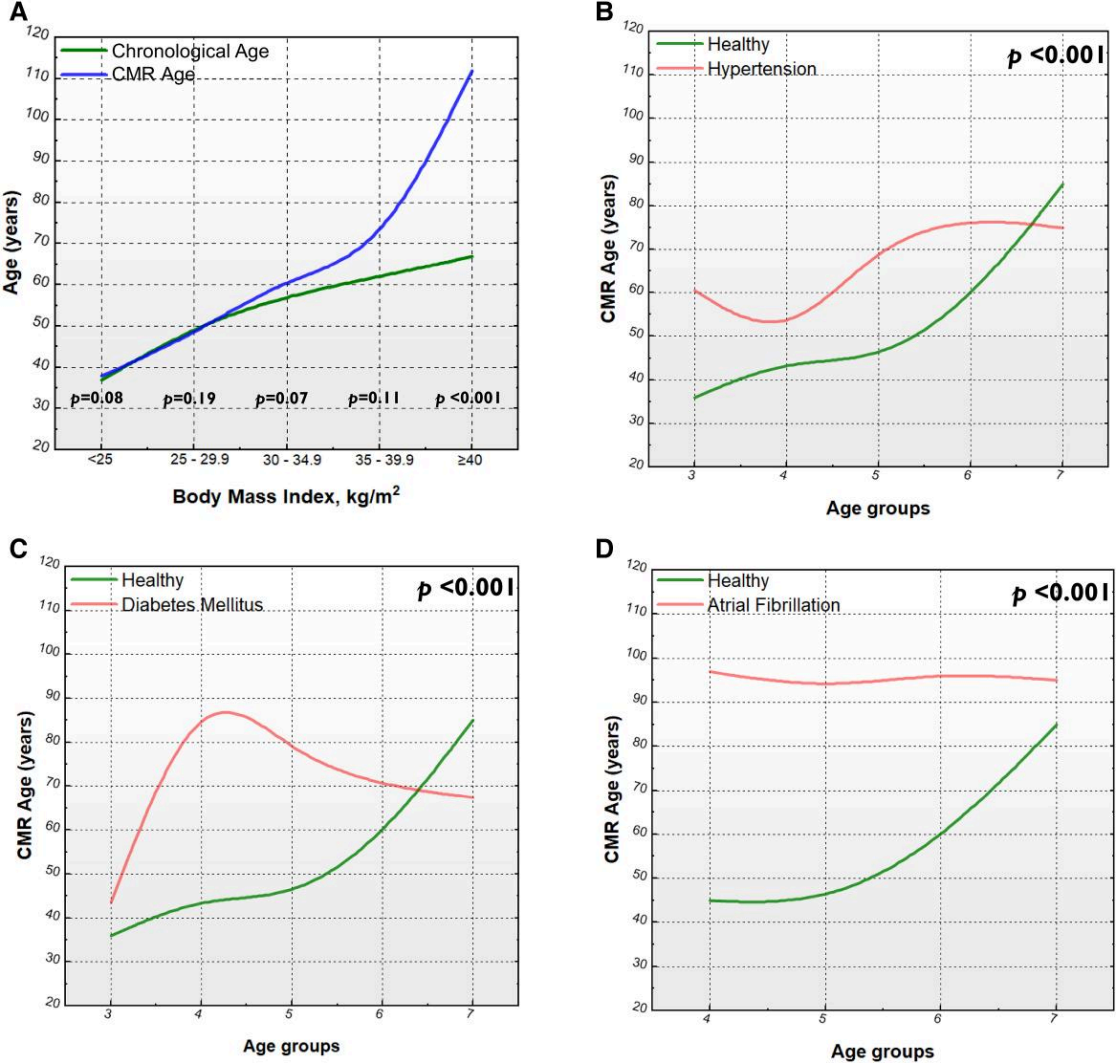
Line graphs demonstrating cardiac magnetic resonance-derived functional age in different disease states. (*A*) The differences between chronological age and cardiac magnetic resonance-derived functional heart age according to the body mass index as per the World Health Organization criteria. (*B*) The differences between cardiac magnetic resonance-derived functional heart age in healthy subjects and patients with hypertension. (*C*) The differences between cardiac magnetic resonance-derived functional heart age in healthy subjects and patients with diabetes mellitus. (*D*) The differences between cardiac magnetic resonance-derived functional heart age in healthy subjects and patients with atrial fibrillation. The *P*-values are for independent sample *t*-tests.

Normal and overweight patients had similar CMR-derived functional heart age and chronological age. For patients who were obese, CMR-derived functional heart age was higher than the chronological age by 4 years in obesity Class I (*P* = 0.07), 5 years in obesity Class II (*P* = 0.11), and 45 years in obesity Class III (*P* < 0.001; *Figure 4A*).

### Cardiac magnetic resonance-derived functional heart age trends with comorbidities

Cardiac magnetic resonance-derived functional heart age was significantly higher in patients with hypertension from Groups 3 to 6 and lower in Group 7 (*P* < 0.001) than in healthy individuals of the same age group (*Figure 4B*). Moreover, compared with healthy controls, patients with DM had an initial 7–8-year exaggeration in Group 3, rising steeply to around 40–56 years in Group 4 before the difference decreased gradually to 20–30 years in Group 5, 18 years in Group 6 and then −21 years in Group 7 (*P* < 0.001; *Figure 4C*). In patients with AF, CMR-derived functional heart age was significantly higher than healthy controls in all age-matched groups (*P* < 0.001; *Figure 4D*).

In a subgroup analysis, we included all patients with comorbidities in a composite model. On univariate analysis, LA EDV, LA ESV, LA GLS, LV mass, and RA ESV significantly correlated positively, and LA EF and RA EF correlated negatively with the composite group (all *P* < 0.001). On stepwise multi-variable linear regression analysis, only LA ESV, LV mass, and LA GLS remained associated in the step increase with comorbidities (see Supplementary material online, *Table S6*).

## Discussion

In this multi-centre, international, cross-sectional cohort study, we sought to investigate the structural and functional alterations in the heart and their link to pre-mature cardiovascular ageing. We used AI-derived CMR analysis to develop a model to estimate functional heart age and revealed which LA, LV, RA, and RV parameters increased or decreased with healthy ageing. Moreover, we highlighted the CMR biomarkers associated with accelerated cardiovascular ageing in obesity, hypertension, DM, and AF. We demonstrated that unhealthy individuals generally had higher CMR-derived functional heart age than their chronological age by 4.6 years, 4 years if in obesity Class I, 5 years if in obesity Class II, and 45 years if in obesity Class III. Furthermore, we showed that patients with hypertension are pre-maturely older by CMR until the seventh decade of life is reached; patients with DM initially had an exaggerated CMR-derived functional heart age in the third decade of life, rising steeply in the fourth decade of life before decreasing gradually in the fifth and sixth decades of life; patients with AF had a significantly higher CMR-derived functional heart age in all decades of life.

Our findings are consistent with previous studies proposing methods to estimate biological heart age and predict clinical outcomes, including DNA methylation and telomere length,^25^ race and sex,^26^ and deep standard Doppler echocardiography,^27^ and deep learning models using electrocardiogram,^28^ cardiac computed tomography and CMR.^29^ In 35 heart donor patients, Pavanello *et al.*^25^ demonstrated that the biological age of heart tissues was 12 years younger than the donor's chronological age. Moreover, in the Coronary Artery Risk Development in Young Adults (CARDIA) study, utilizing the non-laboratory-based Framingham 10-year cardiovascular disease risk calculator, Zmora *et al.*^26^ found that risk factors such as smoking status, education, BMI, systolic blood pressure, DM, and physical activity significantly influenced the difference between biological and chronological heart age. In our multi-centre study, patients with cardiovascular risk factors had a CMR-derived functional heart age of 4.6 years higher than chronological age. Our study also corroborates the literature by demonstrating that patients with DM, hypertension, AF, and obesity exhibit a significantly higher CMR-functional heart age than their chronological age. These findings are consistent with previous reports suggesting that these modifiable risk factors contribute to pre-mature cardiovascular ageing, likely due to their increased strain on the cardiovascular system.

Healthy ageing is associated with increased LV mass-to-volume ratio (concentricity) and decreased ventricular dimensions.^30^ Structural changes such as increased aortic root diameter, LV wall thickness, and altered LV diastolic filling are also associated with ageing.^20^ Left ventricular remodelling, commonly observed in cardiac diseases, is also associated with ageing, with men experiencing increased LV mass and both sexes experiencing decreased EDV and increased mass-to-volume ratio over time.^30^ Obesity is associated with an increased risk of HF, mainly due to obesity-related metabolic, inflammatory, and hormonal changes.^6^ Hypertension contributes to LV hypertrophy and can exacerbate cardiac remodelling, especially when present alongside obesity and DM.^31^ On the other hand, DM, particularly when poorly controlled, is associated with an increased likelihood of HF and can worsen outcomes in patients with an established diagnosis of HF.^32^ Additionally, the co-occurrence of DM and hypertension can lead to deterioration of left atrioventricular coupling and LA function, when compared with individuals with DM only.^33^

### Effects of ageing by cardiac chamber

#### Right atrium

In this study, the healthy right atrium almost linearly increases in volume during both systole (22–40 mL) and diastole (50–73 mL) with ageing. The increase in volume is associated with a complex change in the right atrial EF, with the initial rise in the second decade of life before a progressive decrease that accelerates in the seventh and eighth decades of life. Thus, it starts from 52%, peaks at 55%, and reaches the nadir of 45%. The curve depicting the right atrial global longitudinal strain is the mirror opposite of the right atrial EF. These changes imply a natural decline in the efficiency of the right atrium as both a reservoir and, more importantly, as a pump from the end of the second decade of life. The decline accelerates after the seventh decade of life. Similar trends were observed in previous studies, demonstrating that older people exhibit larger RA volumes and lower EF^34^ than younger individuals.

On the other hand, the unhealthy cohort in this study had higher right atrial volumes at younger ages, with a slight decline as the unhealthy adults continued to age. Remarkably, the right atrial EF curve appears to have a less steep decline as the ageing process progresses, which may well be a reflection of RA compliance. The mirror effect between the right atrial EF curves and that of the right atrial global longitudinal strain is also present in unhealthy adults.

#### Left atrium

In this study, LA EDV increases throughout life with steep acceleration of that increase in the third and the eighth decades; the volume rises from just <50 mL during the teens to a peak of 90 mL after the age of 75 years. During systole, the volume increases progressively with a slight acceleration in the seventh and eighth decades of life, from around 15 to 40 mL. The LA volumes in the unhealthy cohort are slightly higher early in life but follow a similar trajectory, rising as the unhealthy adult ages. These changes are associated with a progressive and steep decline in the LA EF from 66 to 55%, with the decline becoming steeper after the fifth decade. The LA GLS generally declines with the LA EF in healthy individuals. However, the time course differs in that there are three steep rises in the second, fifth, and eighth decades of life. The LA EF and the LA GLS curves in the unhealthy cohort generally track those seen in healthy individuals but without the stepwise increases. Therefore, LA grows in volume and declines in efficiency as a pump with ageing, confirming the findings of the World Alliance Societies of Echocardiography study in which LA volumes rose incrementally and LA EF reduced with ageing.^34^

#### Right ventricle

In this study, RV volume oscillates in end-systole and end-diastole around 25–30 and 65–78 mL, respectively. However, the oscillation appears to be more pronounced in end-diastole, with two peaks in the third and seventh decades and a deep trough in the fifth decade. In end-systole, there appears to be an overall downward trend from teens to the eighth decade. Consequently, one could easily understand that RV EF tends to rise throughout life. In contrast, RV GLS drops overall in the presence of two peaks coincident with the peaks of the RV ESV curve with a nadir in the fifth decade.

The physiological changes we observed in the RV due to ageing remain complex. Our results confirm the findings of previous studies, which highlighted the decrease in RV EDV, ESV, and GLS and the increase in RV EF^30^ as we grow older. These alterations could reflect a compensatory mechanism of the heart to maintain CO despite the reduction in cardiac volumes.

#### Left ventricle

In healthy individuals in this study, LV volumes in systole and diastole change throughout life, tending to decline in both systolic and diastolic volumes, resembling that seen in the RV. In diastole, the volume initially increases from its level in the teens towards a peak in the third decade before decreasing progressively from 130 mL to a nadir of 115 mL in the eighth decade. The curve’s descent is interrupted by a small peak in the seventh decade at 120 mL. A similar trend is seen in systole, with a peak of 50 mL in the third decade and a nadir of 35 mL in the eighth decade. Our results mirror those of previous studies demonstrating the age-associated decline of LV EDV, ESV, and SV.^30,35^ Consequently, LV EF rises slowly from the age of 20 until the seventh decade, when it is around 63%, before undergoing a steep rise to 70% in the eighth decade. This compensatory increase in LV EF with ageing to maintain CO despite the reduction in cardiac volumes confirms findings by previous studies.^35^

Left ventricle GLS also rises throughout life, opposite to the behaviour of the RV GLS. The behaviour of these measurements in the unhealthy cohort shows a general and significant increase in the volumes in systole and diastole with lower LV EF in the first decades and a loss of the rise in the EF after the seventh decade.

In health, LV mass slowly declines with ageing from the teens until the sixth decade, where it rises steeply towards its peak at old age (over 70 years). This modest change reflects the physiological remodelling due to normal adaptive compensation in response to ageing, seen similarly by previous studies.^30,36^ The time course of LV mass is very different in unhealthy adults. It initially rises, followed by a declining curve, reaching a nadir in the fifth decade. It then rises again to a second peak that remains lower than the first, though still higher than the peak in healthy old adults.

Left ventricle PER increases slightly from 400 to around 420 mL/s by the age of 20 years before it declines progressively to a nadir of 250 mL/s by the sixth decade. It then rises again as the ageing process continues to around 350 mL/s in the eighth decade. In unhealthy adults, the initial peak at 20 years is much higher, at just below 450 mL/s, before progressively dropping to around 350 mL/s in the eighth decade. The trend of LV PFR in healthy and unhealthy adults with ageing was similar, with an initial ascent to a peak around 30 years before progressive descent, with the two curves almost meeting at 400 mL/s. However, the healthy adult’s curve starts from 450 mL/s to a peak of just over 500 mL/s, while the unhealthy adult curve starts at the peak of 600 mL/s before progressively declining.

### Clinicopathological implications

Our study endorses the concepts laid out by recent work on the non-invasive techniques for tracking biological ageing of the cardiovascular system^29^ and the nonlinear nature of ageing, especially around 40 and 60 years of age.^12^ However, understanding cardiovascular morphological changes associated with ageing remains vague and ill-described. This study is the first to attempt to develop a simple CMR model to estimate functional heart age. Increased LA volume is an integral component of diagnosing HF with preserved EF (HFpEF).^37^ The guidelines recognize the impact of the development of AF on the LA volume; they suggest that a LA volume above 55 mL/m^2^ is a significant marker for increased risk of AF.^38^ Our findings raise the question of whether these thresholds need to be adjusted according to the patient’s age. The LV EF rises in healthy people with age, becoming particularly striking beyond the age of 60 years. Present guidelines set a dichotomous LVEF threshold of 50%, above which the LVEF, and therefore the systolic function, is considered preserved. In the presence of HF symptoms and signs, an LVEF >50% would define HFpEF, whilst an LVEF <50% would define HF with mid-range EF or HF with reduced EF.

Elderly patients beyond the age of 75 years are under-represented in HF trials.^39^ Based on our observations and those of others, we call upon the cardiology community to better define HF phenotypes with age to potentially enable the very elderly population to access effective, evidence-based treatment.

### Limitations

By necessity, the study is retrospective in design and does not longitudinally follow individual patients but rather takes a snapshot of different age groups in a large, multi-centre, heterogeneous cohort. Though a longitudinal design would be ideal, it would take more than a lifetime to conduct. The study is, therefore, potentially susceptible to survivor bias, which might blunt the effect of comorbidities at older ages, as these patients have survived despite exposure. The duration of comorbidity is also not measured, potentially creating heterogeneity in the duration of exposure in the unhealthy cohort. Other more nuanced features of comorbidity, including diet, socioeconomic status, and exercise, were not assessed, though we might expect them to manifest as disease, which would result in reduced differences between healthy and unhealthy controls. There may also be heterogeneity introduced to the unhealthy group from medical treatments. Finally, there might be a degree of selection bias as we removed cases with missing annotations, substantial contouring failure of cardiac chambers, image artefacts, or misplaced slice position; however, this was limited to only 1% of cases.

## Conclusions

This study highlights the time course of structural and physiological changes in the heart during healthy and unhealthy ageing, which remains complex. Many accepted measures of cardiac function, including EF, may require age adjustment to factor in reduced compliance of the heart during healthy ageing. By demonstrating the discrepancy in cardiac and functional age, CMR-derived functional heart age might highlight patients in need of risk factor interventions and help simplify concepts for patients.

## Lead author biography


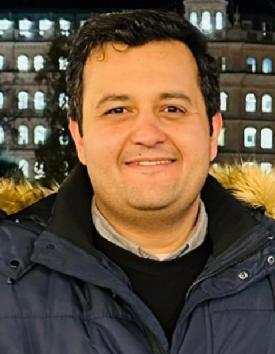


Dr Hosamadin S. Assadi is a clinical PhD fellow at the University of East Anglia and an Honorary Scientist at Norfolk and Norwich University Hospitals NHS Foundation Trust. With a Bachelor of Medicine, Bachelor of Surgery background and an MRes degree in Cardiovascular Medicine from the University of Sheffield, he specializes in advanced cardiovascular magnetic resonance (CMR) imaging post-processing methods, including volumes, scar, fibrosis and 4D flow CMR. His research focuses on developing advanced groundbreaking CMR techniques using artificial intelligence (AI). Those innovative techniques will lead to more efficient diagnoses, better treatment decisions, and improved outcomes for patients with cardiovascular disease.

## Supplementary Material

oeaf032_Supplementary_Data

## Data Availability

The datasets generated and analysed during the current study are not publicly available. Access to the raw images of patients is not permitted since specialized post-processing imaging-based solutions can identify the study patients in the future. Data are available from the corresponding author upon reasonable request.
