# Supplementary material for: Cardiovascular magnetic resonance imaging markers of ageing: a multi-centre, cross-sectional cohort study
Source: Eur Heart J Open. 2025 May 2;5(3):oeaf032. doi: 10.1093/ehjopen/oeaf032 (PMC12045662; doi:10.1093/ehjopen/oeaf032)
Supplement: oeaf032_Supplementary_Data [file oeaf032_supplementary_data.zip › SupplementalAppendix_EHJ.v5.pdf]

Cardiac MRI Markers of Ageing: A Multicentre, Cross-sectional Cohort Study

Supplemental material

Table of Contents

Cardiac MRI Markers of Ageing: A Multicentre, Cross-sectional Cohort Study....1

Supplemental material....1

Supplemental Methods....2

Patient and public involvement....2

CMR protocol....3

Further cardiac-age model development details....4

Supplemental Results....5

Preliminary external validation....5

Supplemental Figures....6

Supplemental Figure 1....6

Supplemental Tables....7

Supplemental Table 1....7

Supplemental Table 2....8

Supplemental Table 3....9

Supplemental Table 4....11

Supplemental Table 5....13

Supplemental Table 6....14

Supplemental Methods

Patient and public involvement

The engagement of patients and the public was initiated at the project's inception through Norfolk and Suffolk Primary and Community Care Research Office (https://nspccro.nihr.ac.uk/working-with-us/public-patient-and-carer-voice-in-research). The patient and public involvement (PPI) panel helped to make the study protocol patient-friendly. The PPI group also provided insight into the study's design of patient information sheets and agreed that the research would produce open-access research papers available to all to read.

CMR protocol

1. Norwich: 1.5-T Magnetom Sola system by Siemens Healthineers (Erlangen, Germany). Cine CMR acquisitions were performed using a cardiac-gated balanced steady-state free precession (bSSFP) sequence during end-expiratory breath-hold. The CMR protocol included baseline survey images and cine sequences. Following planning sequences, four-chamber cine images were acquired, followed by a stack of short-axis cine images covering apex to base.
2. Leeds: 1.5-T Ingenia system by Philips Healthcare (Best, the Netherlands). Cine CMR acquisitions were performed using a bSSFP single-slice breath-hold sequence, including baseline survey images and standard cines.
3. Sheffield: 1.5-T HDx system by GE Healthcare (Chicago, Illinois, USA). Cine images were acquired during end-expiratory breath hold with a bSSFP single-slice breath-hold sequence, and baseline survey images and standard cines were included.
4. Cordoba: 3.0-T Achieva system by Philips Healthcare (Best, the Netherlands), 1.5-T Magnetom Essenza system by Siemens Healthineers (Erlangen, Germany), and 1.5-T Ingenia system by Philips Healthcare (Best, the Netherlands). Cine CMR acquisitions were performed using a bSSFP single-slice breath-hold sequence, including baseline survey images and standard cines.
5. Singapore: 3.0-T Ingenia by Philips Healthcare (Best, the Netherlands) and 1.5-T Magneton Aera by Siemens Healthineers (Erlangen, Germany). Cine CMR acquisitions were performed using a bSSFP single-slice breath-hold sequence and retrospective electrocardiographic gating. The CMR protocol included baseline survey images and cine sequences, cine long-axis images at two, three, and four-chamber views, followed by a stack of short-axis cine images covering apex to base.

Further cardiac-age model development details

As seen in **Supplemental Table 4**, the two most linearly correlated parameters are LA ESV and LA EDV. Due to the high degree of collinearity observed in the population, we selected LA ESV, as it is a superior marker of left ventricular afterload. Additionally, LA EF exhibited a negative correlation with ageing and was selected as it is a functional parameter independent of body surface area, unlike other parameters that anthropometric factors may influence. Moreover, we prioritised LA EF over LA GLS because it demonstrated a more consistent decline with ageing. While LA EF was associated with a progressive and steep decline, LA GLS displayed variability with steep rises observed in the 2nd, 5th, and 8th decades of life, complicating its integration into the CMR functional heart age model equation (**Figure 2**).

Supplemental Results

Preliminary external validation

In a subanalysis, we applied the above equations to an independent healthy validation cohort (*n* = 25) free of any metabolic, cardiovascular, or respiratory disease (mean body mass index: 24.7 kg/m^2) to estimate functional heart age (**Supplemental Table 5**). The correlation coefficient of the CMR-derived functional heart age and chronological age was (r = 0.695, 95% CI: 0.413 to 0.855, p<0.001). On Bland-Altman analysis, the mean difference between CMR-derived age and chronological age was (*bias*: -7.1%, 95% CI: -26.5−12.4%, p=0.460) (**Supplemental Figure 1**).

Supplemental Figures

Supplemental Figure 1. Bland–Altman plot demonstrating the degree of agreement between chronological age and CMR-derived age in the external validation cohort (n = 25).

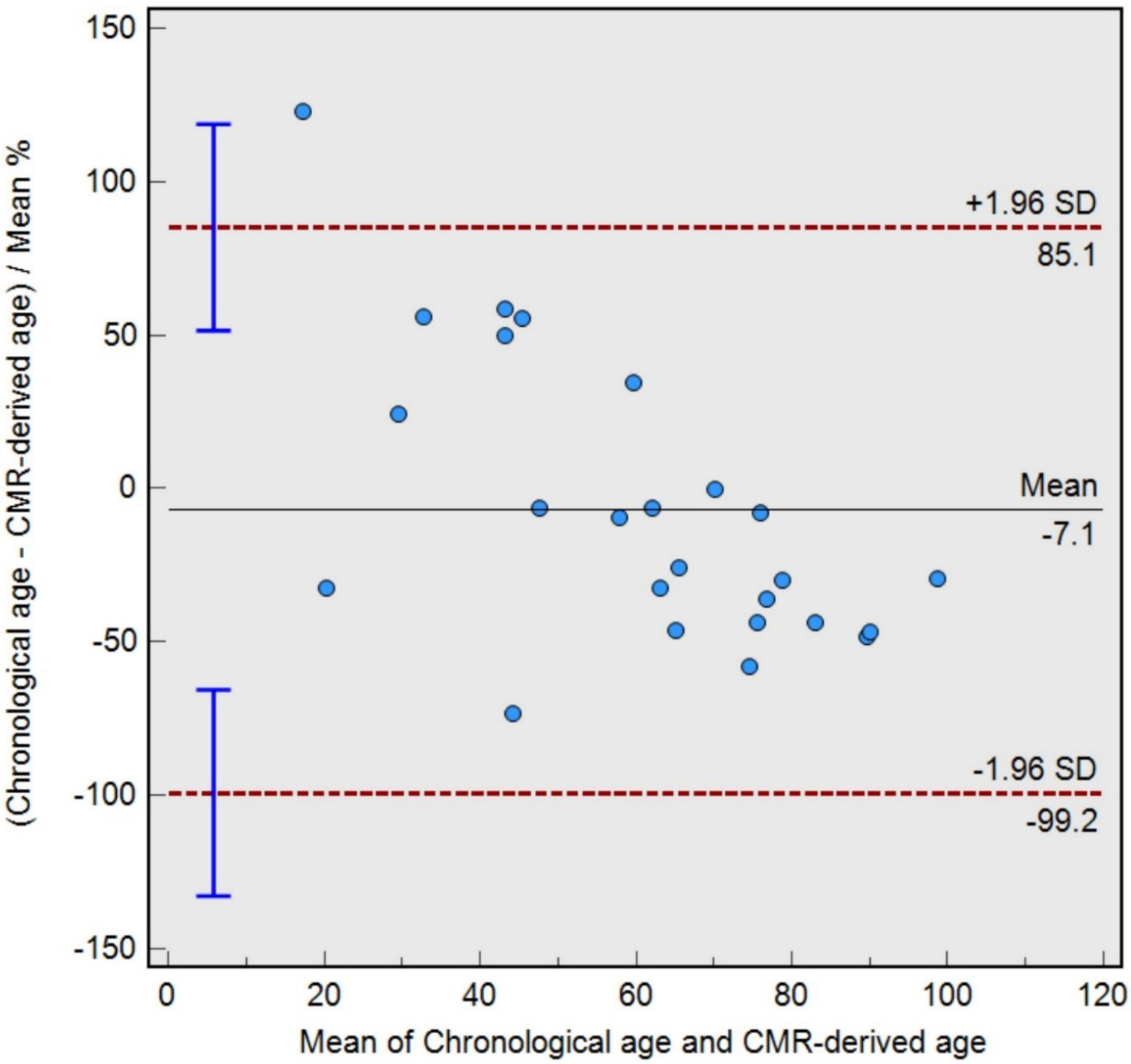

Supplemental Tables

Supplemental Table 1. Cine CMR acquisition parameters for the five centres.

Centre	Norwich	Leeds	Sheffield	Cordoba			Singapore	
Vendor	Siemens	Philips	GE	Philips	Siemens	Philips	Philips	Siemens
Magnetic field strength	1.5-T	1.5-T	1.5-T	3.0-T	1.5-T	1.5-T	3.0-T	1.5-T
Pulse sequence	bSSFP	bSSFP	bSSFP	bSSFP	SSFP	bSSFP	bSSFP	bSSFP
Cine images								
TR/TE (ms)	2.71/1.13	2.72/1.36	3.7/1.6	2.8/1.4	58/1.5	2.8–3.2/1.4–1.6	2.8/1.4	3.4/1.3
Flip angle (°)	80	60	60	45	58	60	45	72
Field of view (mm^2)	360 × 289	320 × 420	380 × 380	320 × 320	340 × 280	300 × 300	320 × 320	320 × 260
Slice thickness, mm	8	8	8	8	8	8	8	8
Cardiac phases	30	30	30	30	25	30	30	30

Supplemental Table 2. Interpolated blunted age for higher (>50 mL) and lower (<15 mL) extremes of left atrial end-systolic volume.

**Left atrial end-systolic volume >50 mL**	**Age (years)**
50	90
80	92
100	94
150	96
200	98

**Left atrial end-systolic volume <15 mL**	**Age (years)**
15	10
10	9
5	8
4	7

Supplemental Table 3. Study demographics and CMR characteristics of non-European *versus* European healthy participants.

**Demographics**	**Non-European (*n*=96)**	**European (*n*=95)**	***p*-value**
Age, years	38.5 (31−52)	26 (17−42)	<0.0001
Female sex, n (%)	52 (54%)	53 (56%)	0.822
Body mass index, kg/m^2	21.1 (20−22)	21.7 (20−23)	0.037
**Left heart**			
Left atrial end-diastolic volume, mL	71 (59−80)	64 (47−84)	0.093
Left atrial end-systolic volume, mL	25 (20−31)	22 (15−29)	0.050
Left atrial stroke volume, mL	44 (38−50)	41 (31−53)	0.142
Left atrial ejection fraction, %	65 (60−68)	66 (61−71)	0.137
Left atrial global longitudinal strain, %	-27 (-31−-24)	-29 (-35−-24)	0.053
Left ventricular end-diastolic volume, mL	121 (107−140)	133 (110−162)	0.013
Left ventricular end-systolic volume, mL	45 (39−55)	50 (42−61)	0.032
Left ventricular stroke volume, mL	75 (66−83)	80 (67−97)	0.038
Left ventricular mass, g	79 (66−95)	94 (75−115)	0.001
Left ventricular ejection fraction, %	62 (59−65)	62 (58−65)	0.546
Left ventricular peak ejection rate, mL/sec	354 (311−419)	417 (332−468)	0.001
Left ventricular peak filling rate, mL/sec	437 (389−523)	469 (380−605)	0.06
Left ventricular cardiac output, mL/min	4479 (4091−5259)	5179 (4307−6338)	0.009
Left ventricular global longitudinal strain, %	-21 (-22−-19)	-22 (-25−-20)	0.001
**Right heart**			
Right atrial end-diastolic volume, mL	65 (55−74)	65 (52−79)	0.834
Right atrial end-systolic volume, mL	30 (24−37)	28 (23−41)	0.922
Right atrial stroke volume, mL	34 (29−40)	33 (26−43)	0.374
Right atrial ejection fraction, %	54 (48−59)	52 (47−59)	0.471
Right atrial global longitudinal strain, %	-29 (-32−-25)	-27 (-31−-24)	0.033
Right ventricular end-diastolic volume, mL	66 (53−85)	73 (62−89)	0.012

Right ventricular end-systolic volume, mL	25 (18−35)	28 (22−37)	0.069
Right ventricular stroke volume, mL	40 (33−50)	45 (38−56)	0.004
Right ventricular ejection fraction, %	62 (58−67)	62 (58−66)	0.746
Right ventricular cardiac output, mL/min	2482 (1997−3148)	2789 (2428−3761)	0.008
Right ventricular global longitudinal strain, %	-32 (-36−-29)	-32 (-36−-29)	0.819

Data are given as median (25th percentile−75th percentile), a Mann-Whitney test.

Supplemental Table 4. Correlation coefficient of the CMR functional variables associated with ageing in the healthy cohort.

	Healthy cohort (*n*=191)	
**Variable**	**rho**	***p*-value**
Left atrial end-diastolic volume, mL	0.348	**<0.0001**
Left atrial end-systolic volume, mL	0.365	**<0.0001**
Left atrial stroke volume, mL	0.266	**0.0002**
Left atrial ejection fraction, %	-0.243	**0.0007**
Left atrial global longitudinal strain, %	0.245	**0.0006**
Left ventricular end-diastolic volume, mL	-0.07	0.339
Left ventricular end-systolic volume, mL	-0.099	0.172
Left ventricular stroke volume, mL	-0.023	0.757
Left ventricular mass, g	-0.068	0.349
Left ventricular ejection fraction, %	0.105	0.150
Left ventricular peak ejection rate, mL/sec	-0.203	**0.005**
Left ventricular peak filling rate, mL/sec	-0.32	**<0.0001**
Left ventricular cardiac output, mL/min	-0.201	**0.008**
Left ventricular global longitudinal strain, %	0.173	**0.017**
Right atrial end-diastolic volume, mL	0.274	**0.0001**
Right atrial end-systolic volume, mL	0.288	**0.0001**
Right atrial stroke volume, mL	0.141	0.057
Right atrial ejection fraction, %	-0.191	**0.009**
Right atrial global longitudinal strain, %	0.019	0.798
Right ventricular end-diastolic volume, mL	-0.148	**0.041**
Right ventricular end-systolic volume, mL	-0.22	**0.002**
Right ventricular stroke volume, mL	-0.068	0.350
Right ventricular ejection fraction, %	0.268	**0.002**
Right ventricular cardiac output, mL/min	-0.154	**0.043**

Right ventricular global longitudinal strain, %	-0.173	**0.017**

Bold values denote statistical significance. Healthy includes individuals with normal body mass index (BMI) <25 kg/m^2 and free of any metabolic, cardiovascular and respiratory disease.

Supplemental Table 5. Study demographics of the external validation cohort (n = 25).

**Demographics**	**Healthy (*n*=25)**
Age, years	55 ± 16
Female sex, ***n*** (%)	9 (36)
Body mass index, kg/m^2	24.7 ± 3
Hyperlipidaemia, ***n*** (%)	0 (0)
Hypertension, ***n*** (%)	0 (0)
Diabetes mellitus, ***n*** (%)	0 (0)
Atrial fibrillation, ***n*** (%)	0 (0)
Myocardial infarction, ***n*** (%)	0 (0)

Healthy includes individuals with normal body mass index (BMI) <25 kg/m^2 and free of any metabolic, cardiovascular and respiratory disease.

Supplemental Table 6. Stepwise multivariable linear regression analysis.

Dependent Y	Composite model					
**Least squares multiple regression**						
**Method**	Stepwise					
**Enter variable if *p*<**	0.001					
**Remove variable if *p*>**	0.01					
**Sample size**	540					
**Coefficient of determination *R2***	0.2497					
***R2*-adjusted**	0.2455					
**Multiple correlation coefficient**	0.4997					
**Residual standard deviation**	0.9752					
**Regression Equation**						
**Independent variables**	Coefficient	Std. Error	t	*p*-value	rpartial	rsemipartial
**(Constant)**	0.3906					
**LA end-systolic volume**	0.01258	0.00231	5.444	<0.0001	0.2289	0.2037
**LV mass**	0.007423	0.001337	5.55	<0.0001	0.2331	0.2076
**LA global longitudinal strain**	0.02073	0.005836	3.553	0.0004	0.1517	0.1329
**Variables not included in the model**						
**LA end-diastolic volume index**						
**RA end-systolic volume**						
**LA ejection fraction**						
**RA ejection fraction**						
**Analysis of Variance**						
**Source**	DF	Sum of Squares	Mean Square			
**Regression**	3	169.643	56.5477			
**Residual**	536	509.757	0.951			
**F-ratio**	59.4588					
**Significance level**	*p* <0.0001					
