# Supplementary figures and images for: Cardiovascular magnetic resonance imaging markers of ageing: a multi-centre, cross-sectional cohort study
Source: Eur Heart J Open. 2025 May 2;5(3):oeaf032. doi: 10.1093/ehjopen/oeaf032 (PMC12045662; doi:10.1093/ehjopen/oeaf032)

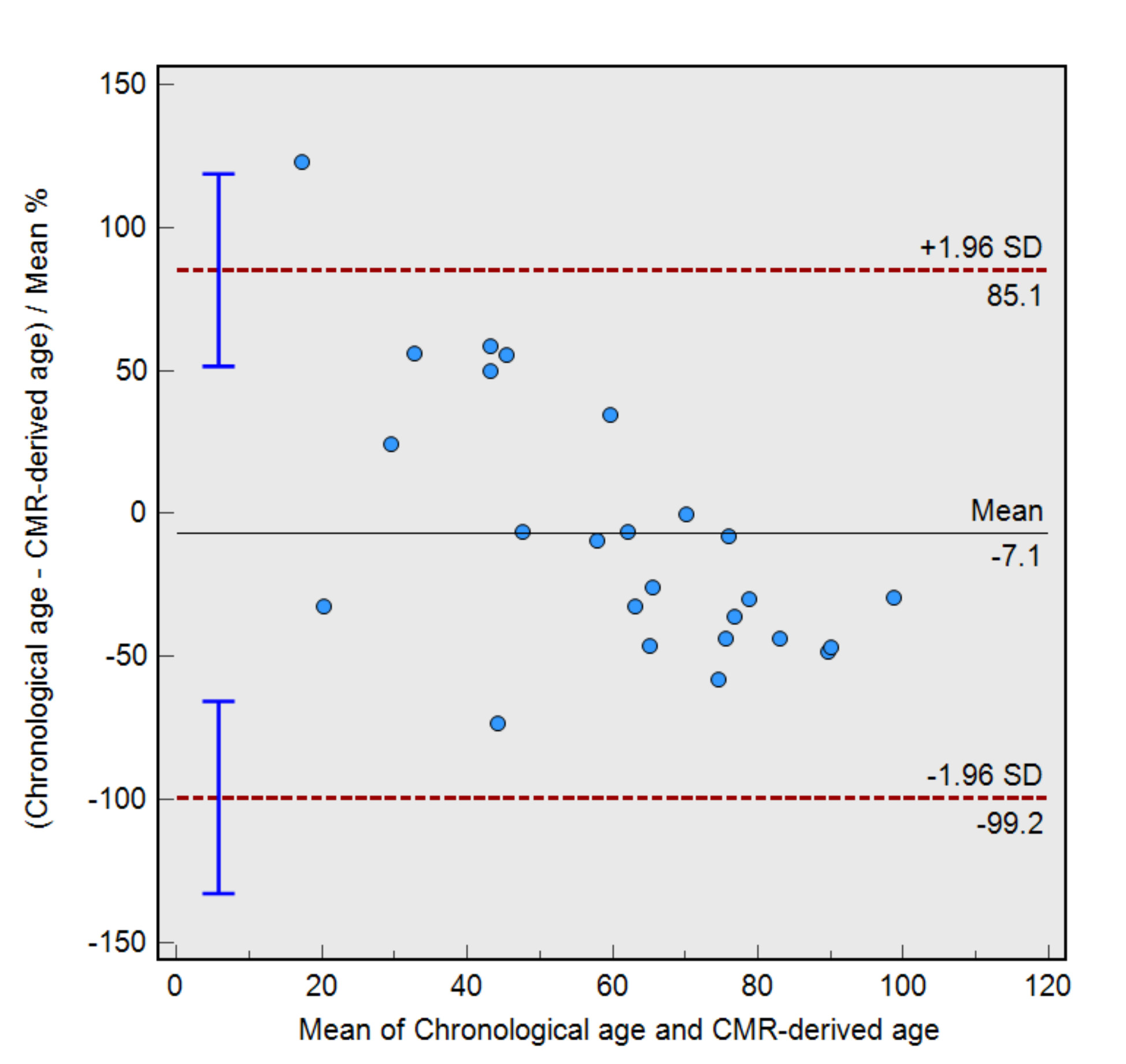

Supplement: oeaf032_Supplementary_Data [file oeaf032_supplementary_data.zip › Suppl Figure 1.jpg]
